# Three out of four working-age patients have fulfilled expectations towards paid employment six months after total hip or knee arthroplasty: a multicentre cohort study

**DOI:** 10.1007/s00296-023-05437-9

**Published:** 2023-08-29

**Authors:** Tamara Kamp, Martin Stevens, Thea P. M. Vliet Vlieland, Rob G. H. H. Nelissen, Sandra Brouwer, Maaike G. J. Gademan, on behalf of the Longitudinal Leiden Orthopaedics Outcomes of Osteoarthritis Study (LOAS) Group

**Affiliations:** 1 Department of Orthopaedics, University Medical Center Groningen, University of Groningen, P.O. Box 30.001, 9700 RB Groningen, The Netherlands; 2 Department of Health Sciences, Community and Occupational Medicine, University Medical Center Groningen, University of Groningen, Groningen, The Netherlands; 3 Department of Orthopaedics, Leiden University Medical Center, Leiden, The Netherlands; 4 Department of Clinical Epidemiology, Leiden University Medical Center, Leiden, The Netherlands

**Keywords:** Arthroplasty, Replacement, Knee, Hip, Work, Motivation, Patient Satisfaction

## Abstract

To investigate factors associated with fulfilment of expectations towards paid employment after total hip/knee arthroplasty (THA/TKA). Cohort study including preoperatively employed patients aged 18–64 scheduled for THA/TKA. Expectations were collected preoperatively, and 6 and 12 months postoperatively with the paid employment item of the Hospital-for-Special-Surgery Expectations Surveys (back-to-normal = 1; large improvement = 2; moderate improvement = 3; slight improvement = 4; not applicable = 5). Patients scoring not applicable were excluded. Fulfilment was calculated by subtracting preoperative from postoperative scores (< 0: unfulfilled; ≥ 0: fulfilled). Multivariable logistic regression analyses were conducted separately for THA/TKA at 6 and 12 months postoperatively. Six months postoperatively, 75% of THA patients (n = 237/n = 316) and 72% of TKA patients (n = 211/n = 294) had fulfilled expectations. Older age (TKA:OR 1.08, 95% CI 1.01–1.15) and better postoperative physical functioning (THA:OR 1.10, 95% CI 1.06–1.14; TKA:OR 1.03, 95% CI 1.01–1.06) increased the likelihood of fulfilment. Physical work tasks (THA:OR 0.12, 95% CI 0.03–0.44), preoperative sick leave (TKA:OR 0.33, 95% CI 0.17–0.65), and difficulties at work (THA:OR 0.10, 95% CI 0.03–0.35; TKA:OR 0.41, 95% CI 0.17–0.98) decreased the likelihood of fulfilment. Twelve months postoperatively similar risk factors were found. Three out of four working-age THA/TKA patients had fulfilled expectations towards paid employment at 6 months postoperatively. Preoperative factors associated with fulfilment were older age, mental work tasks, no sick leave, postoperative factors were better physical functioning, and no perceived difficulties at work.

**Supplementary Information:**

The online version contains supplementary material available at 10.1007/s00296-023-05437-9.

## Introduction

Work is a key element of participation and an important determinant of general health, well-being, and quality of life [1]. Studies measuring work-related outcomes have mainly examined first-time return to work (RTW) [2–4], and found that RTW rates varied between 25 and 122% after total hip arthroplasty (THA) and 40–98% after total knee arthroplasty (TKA) within 1–2 years postoperatively [2, 3]. RTW is an important treatment goal for an expanding group of patients undergoing a THA or TKA [2, 3, 5], as 45% of THA and 49% of TKA patients in the Netherlands are of working age [6]. Similar trends are seen in other Western countries [7, 8]. Patients are also confident in reaching this goal, as patients of working-age tend to have high expectations about (returning to) work [5, 9].

Although patient expectations towards paid employment after THA or TKA are high, 11–43% of patients have unfulfilled expectations [9–12]. The few studies investigating fulfilment of expectations among THA or TKA patients suggest that expectations towards paid employment are among the least fulfilled [10, 11]. However, as these studies did not focus on working-age patients [10–12], uncertainties remain about such expectation fulfilment. Moreover, research investigating factors solely associated with fulfilment towards paid employment is so far lacking. The existing studies only incorporate fulfilment towards paid employment as one of multiple factors in their total fulfilment scores [10, 11]. Investigating which factors influence fulfilment of expectations towards paid employment will aid in the design of future target strategies to appropriately address those prone to unfulfillment.

Looking beyond the first time of RTW and focussing on fulfilment of patient expectations towards paid employment is important to gain better understanding of preoperative and postoperative factors associated with a successful RTW process. Therefore, the aim of this study was to identify factors associated with fulfilment of patient expectations towards paid employment for THA or TKA patients at both 6 and 12 months postoperatively. Potential factors include sociodemographic and preoperative/postoperative health- and work-related factors that have previously been linked to RTW [2, 3, 13]. We focussed on fulfilment of expectations towards paid employment at both 6 and 12 months postoperatively, as previous studies indicated that general recovery and recovery of work participation occurs between 6 and 12 months postoperatively [14, 15].

## Methods

### Design and procedure

The study was part of the “Longitudinal Leiden Orthopaedics Outcomes of Osteoarthritis Study” (LOAS), an ongoing, multicentre cohort study [16, 17]. Data collection started in 2012 and patients were recruited at the orthopaedic departments of eight Dutch medical centres (one university hospital and seven regional hospitals). Informed consent was obtained from all patients prior to the study in conformity with the Declaration of Helsinki [18]. For the current study we used data collected preoperatively and at 6 and 12 months postoperatively between 2012 and 2018. Ethical approval was obtained from the Medical Ethics Committee of Leiden University Medical Center (registration no. P12.047; Trial ID NTR3348).

### Population

General inclusion criteria for the LOAS were a diagnosis of osteoarthritis, age 18 or older, being listed for THA/TKA, and sufficient Dutch-language skills to complete the questionnaires. For the current study we selected a subgroup: patients preoperatively employed, aged 18–64, listed for primary THA or TKA, and who completed the preoperative and postoperative item on paid employment.

### Measures

#### Sociodemographic factors

Data were collected preoperatively for the following sociodemographic factors: age (years), sex, and living status (with/without partner).

#### Health-related factors

Health-related factors were gathered by inquiring about body mass index (BMI), comorbidities, ASA-classification, self-reported physical functioning, and health-related quality of life. BMI was derived from preoperative self-reported body height and weight. Comorbidities were measured preoperatively using a 19-item chronic conditions questionnaire developed by the Dutch Central Bureau of Statistics [19]. Comorbidities were categorized as musculoskeletal or non-musculoskeletal (yes/no) [16, 20]. Self-reported osteoarthritis related physical functioning was measured both preoperatively and postoperatively with the validated Hip/Knee Osteoarthritis Outcome Score-Physical function Short form (HOOS-PS/KOOS-PS) [21–23]. The HOOS-PS consists of five items and the KOOS-PS consists of seven items (scale 0–100, higher scores indicating better perceived functioning). Health-related quality of life was measured preoperatively with the Short Form-12 Mental and Physical Component Summary (SF-12 MCS/SF-12 PCS, scale 0–100, higher scores indicating a better health-related quality of life) [24].

#### Work-related factors

Work-related factors, preoperatively collected, were self-employment (yes/no), working hours (h), type of tasks (physical/mental/combination), sick leave one month preoperatively due to hip/knee complaints (yes/no), and expected time to RTW (weeks). Actual time to RTW (weeks) was collected postoperatively. Difficulties at work caused by hip/knee complaints (yes/no) were measured both preoperatively and postoperatively.

#### Preoperative expectations and postoperative fulfilment of expectations towards paid employment

Preoperative expectations towards paid employment were measured using a single question from the validated Hospital for Special Surgery (HSS) hip/knee replacement expectations survey, translated into Dutch [25]. The HSS is an 18-item (THA) or 17-item (TKA) self-administered survey, measuring expectations in the domains of pain, function, activities, and psychological wellbeing. We focused on the “What are your expectations towards paid employment after surgery” item. On a Likert scale, five options were possible: 1 (back to normal), 2 (large improvement), 3 (moderate improvement), 4 (slight improvement), 5 (does not apply). For baseline characteristics of the study population (Table 1), we divided preoperative expectations into “back to normal” (score 1) and “not back to normal” (score 2–4). Patients answering not applicable (score 5) were excluded from the study.

**Table 1:** Baseline study population characteristics of total hip arthroplasty (THA) and total knee arthroplasty (TKA) patients

Variables	THA		TKA	
	Expectation towards paid employment		Expectation towards paid employment	
	Back to normal (N = 345)	Not back to normal (N = 23)	Back to normal (N = 275)	Not back to normal (N = 51)
Sociodemographic factors				
Age (years), mean (SD)	57 (6)	56 (7)	58 (5)	57 (6)
Sex (number of female), % (n)	50% (172)	44% (10)	56% (154)	53% (27)
Partner (yes), % (n)	79% (273)	78% (18)	78% (213)	77% (39)
Health-related factors				
BMI (kg/m^2^), mean (SD)	27 (4)	28 (3)	30 (5)	30 (5)
Comorbidity, % (n)				
Musculoskeletal (yes)	48% (165)	61% (14)	42% (116)	45% (23)
Non-musculoskeletal (yes)	52% (180)	61% (14)	63% (173)	61% (31)
ASA classification, % (n)				
ASA-1	31% (107)	30% (7)	15% (42)	18% (9)
ASA-2	63% (218)	57% (13)	77% (211)	73% (37)
ASA-3	5% (18)	13% (3)	7% (20)	4% (2)
HOOS -PS / KOOS-PS, mean (SD)^a^	45 (17)	43 (19)	41 (16)	38 (17)
Health related QOL, mean (SD)^a^				
SF12 PCS	32 (9)	30 (8)	31 (9)	31 (8)
SF12 MCS	54 (10)	46 (12)	55 (10)	52 (10)
Work-related factors				
Self-employed (yes), % (n)	16% (55)	17% (4)	9% (25)	8% (4)
Working hours preop (per week), mean (SD)	32 (12)	32 (12)	31 (11)	29 (14)
Work tasks % (n)				
Physical	19% (65)	17% (4)	23% (64)	22% (11)
Mental	37% (126)	17% (4)	26% (70)	22% (11)
Both	44% (152)	65% (15)	51% (140)	57% (29)
Sick leave 1 month preop (yes), % (n)	26% (88)	30% (7)	30% (83)	35% (18)
Difficulties at work due to hip/knee (yes), % (n)	84% (289)	96% (22)	91% (250)	94% (48)

Data are represented as mean with standard deviation (SD) or percentages (%) and numbers (n)

Patients were included if they had specified their preoperative expectation and specified either their 6- or 12-month expectation fulfilment question

*BMI* body mass index, *HOOS-PS* Hip injury and Osteoarthritis Outcome Score – Physical function Short form, *KOOS-PS* Knee injury and Osteoarthritis Outcome Score – Physical function Short form, *QOL* quality of life, *SF-12* short form-12, *PCS* Physical Component Summary, *MC S* Mental Component Summary; *preop* preoperatively

^a^All scales ranged from 0 to 100; higher scores indicated better outcomes

Postoperative fulfilment of expectations towards paid employment was used as primary outcome measure. To measure expectation fulfilment, the preoperative HSS questionnaire was modified for use in the LOAS and was composed of the same 5-point Likert scale. The heading of the questionnaire was the only difference: asking to report the “actual status” of the function/activities. Patients were not reminded of their preoperative responses. Postoperative fulfilment of expectations was measured after 6 and 12 months. Fulfilment of expectations was calculated by subtracting preoperative from postoperative HSS scores (≤ – 1: unfulfilled; 0: fulfilled, ≥ 1: exceeded).

### Statistical analyses

Descriptive statistics (mean (SD), n (%)) were used to describe baseline characteristics, for preoperative expectations (“back to normal”, “not back to normal”), separately for THA and TKA patients. Postoperative expectation fulfilment was dichotomized (“unfulfilled” and “fulfilled/exceeded”). The “fulfilled” and “exceeded” groups were combined because the “exceeded” group was very small. Analyses were conducted for fulfilment at both 6 and 12 months postoperatively. The characteristics of patients with unfulfilled and fulfilled expectations were compared using Pearson’s chi-square tests (nominal categorical variables), independent T tests (continuous variables), and chi-square trend tests (ordinal categorical variables; Tables S1 and S2).

To select covariates in the multivariate logistic regression, we performed an univariate test on age, sex, comorbidities, preoperative HOOS-PS/KOOS-PS, postoperative HOOS-PS/KOOS-PS, work tasks, preoperative sick leave and postoperative difficulties at work due to hip or knee complaints (Table S3 and S4). All variables with a p value ≤ 0.15 in the univariate analyses were included in the multivariable regression analyses [26]. Variables were omitted via backward selection, depending on their level of statistical significance (p < 0.05). Preoperative patient expectation was included as control variable. Odds ratios were calculated, including 95% confidence intervals (Tables 3 and 5). All analyses were stratified for THA and TKA. IBM Statistical Package for the Social Sciences (SPSS) version 25.0 was used for analyses.

## Results

**Fig. 1:**
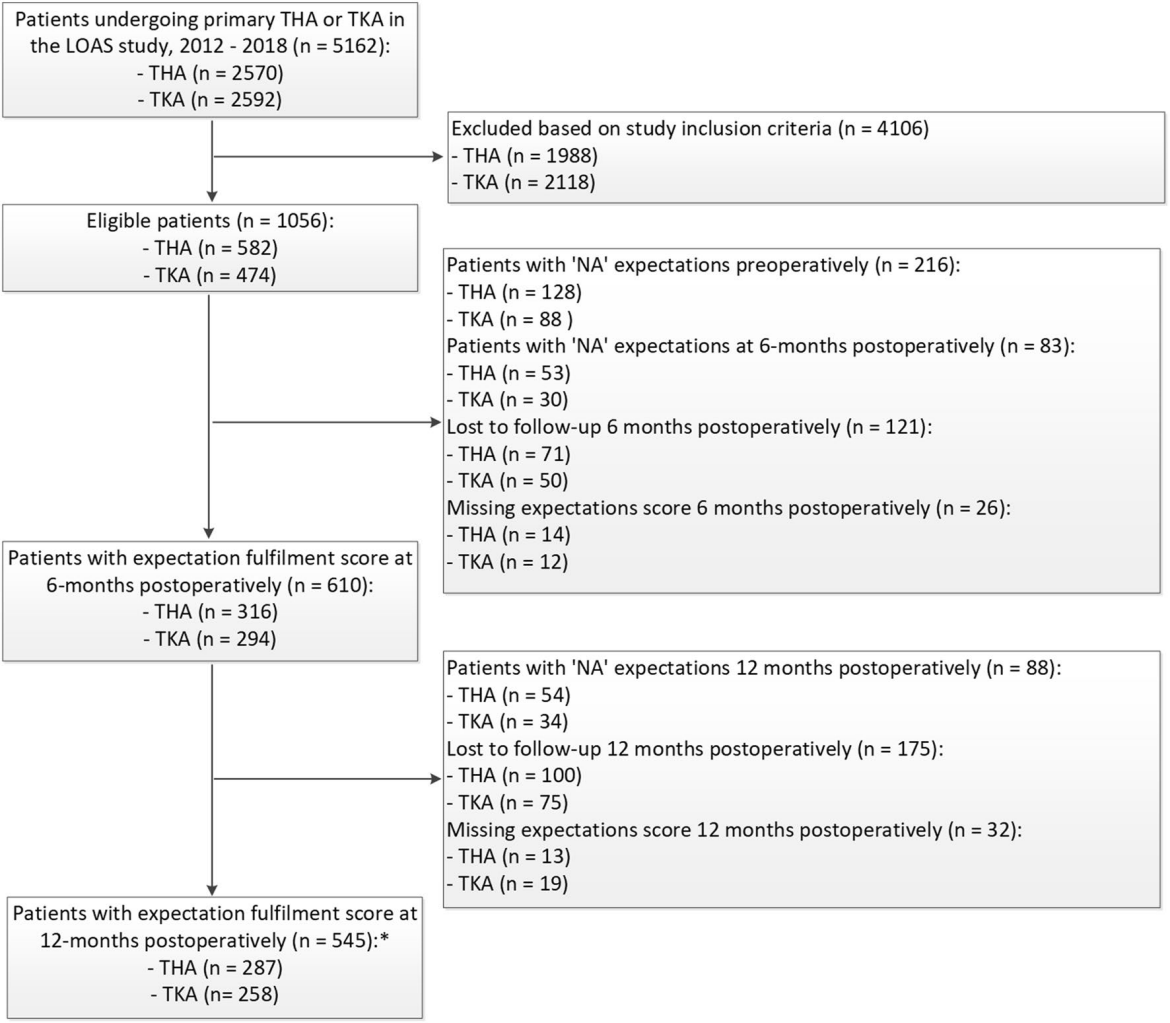
Flowchart study enrolment and follow-up. *From n = 29 THA and n = 36 TKA patients data at 6 months postoperatively was missing, but data at 12 months postoperatively was available. *THA* total hip arthroplasty, *TKA* total knee arthroplasty, *LOAS* Longitudinal Leiden Orthopaedics Outcomes of Osteoarthritis Study, *NA* not applicable

In total, 1056 working-age patients (n = 582 THA, n = 474 TKA) were eligible. Patients answering “not applicable” to the preoperative (n = 128 THA; n = 88 TKA) or postoperative expectation question were excluded (6 months postoperatively: n = 53 THA; n = 30 TKA; 12 months postoperatively: n = 54 THA; n = 34 TKA). The majority of these excluded patients were male (60%) and performed mainly mental work tasks (50%). Eventually, n = 368 THA and n = 326 TKA patients were included. Figure 1 shows a flowchart of the study enrolment and follow-up. Baseline characteristics of the study sample are presented in Table 1.

### Baseline characteristics of THA patients

The THA group consisted of 49% females, mean age 57 years (SD 7). Preoperatively, 94% (n = 345) expected a “back to normal” and 6% (n = 23) expected a “not back to normal” paid employment.

### Baseline characteristics of TKA patients

The TKA group consisted of 56% females, mean age 58 years (SD 5). Preoperatively, 84% (n = 275) expected a “back to normal” and 16% (n = 51) expected a “not back to normal” paid employment.

### Expectation fulfilment of THA patients

Six months postoperatively, 75% (n = 237) of patients had fulfilled expectations and 3% (n = 7) of them exceeded their expectations, increasing 12 months postoperatively to 83% (n = 239) and 4% (n = 10), respectively. Six months postoperatively, 82% of patients with unfulfilled and 96% of patients with fulfilled expectations actually returned to work, increasing 12 months postoperatively to 88% and 98%, respectively. Preoperatively, mean expected time to RTW was 11 weeks for patients with unfulfilled and 7 weeks for patients with fulfilled expectations. Actual mean time to RTW was 12 weeks for patients with unfulfilled and 9 weeks for patients with fulfilled expectations.

Potential risk factors stratified for fulfilment are shown in Tables S1 and S2. Six months postoperatively, five factors were below the cut-off value in the univariate analyses and were therefore used in the multivariate analyses (preoperative HOOS-PS, postoperative HOOS-PS, work tasks, preoperative sick leave, postoperative difficulties at work due to hip complaints; Table S3). Better postoperative physical functioning (HOOS-PS) scores increased the likelihood of fulfilment at 6 months postoperatively (OR 1.10, 95% CI 1.06–1.14). Physical work tasks (reported in 23%; OR 0.12, 95% CI 0.03–0.44) and postoperative difficulties at work due to hip complaints (reported in 41%; OR 0.10, 95% CI 0.03–0.35) decreased the likelihood of fulfilment (Table 2).

**Table 2:** Multivariable logistic regression analyses for the outcome postoperative fulfilment of expectations towards paid employment 6 months after total hip arthroplasty (THA) and total knee arthroplasty (TKA)

Variables	THA		TKA	
	OR	95% CI	OR	95% CI
Age (years)	–	–	1.08	1.01–1.15
HOOS-PS/KOOS-PS postop^a^	1.10	1.06–1.14	1.03	1.01–1.06
Work tasks				
Physical (ref = mental)	0.12	0.03–0.44	–	–
Both (ref = mental)	0.43	0.15–1.26	–	–
Sick leave preop (ref = no)	–	–	0.33	0.17–0.65
Difficulties at work due to hip/knee (ref = no)	0.10	0.03–0.35	0.41	0.17–0.98

Adjusted for preoperative expectation

*HOOS-PS* Hip injury and Osteoarthritis Outcome Score – Physical function Short form, *KOOS-PS* Knee injury and Osteoarthritis Outcome Score—Physical function Short form, *ref* reference category, *Preop* preoperatively, *Postop* postoperatively

^a^Scales ranged from 0 to 100; higher scores indicated better outcomes

Twelve months postoperatively, seven factors were below the cut-off value in the univariate analyses (age, musculoskeletal comorbidity, preoperative HOOS-PS, postoperative HOOS-PS, work tasks, preoperative sick leave, postoperative difficulties at work due to hip complaints; Table S4). Higher age (OR 1.08, 95% CI 1.02–1.15) and better postoperative physical functioning (OR 1.11, 95% CI 1.07–1.14) increased the likelihood of fulfilment. Physical work tasks (OR 0.15, 95% CI 0.04–0.60) and a combination of work tasks (OR 0.14, 95% CI 0.04–0.46) decreased the likelihood of fulfilment (Table 3). Sensitivity analyses showed that a lower proportion of patients aged ≤ 55 had fulfilled expectations compared to those aged ≥ 56.

**Table 3:** Multivariable regression analyses for the outcome postoperative fulfilment of expectations towards paid employment 12 months after total hip arthroplasty (THA) and total knee arthroplasty (TKA)

Variables	THA		TKA	
	OR	95% CI	OR	95% CI
Age (years)	1.08	1.02–1.15	–	–
HOOS-PS / KOOS-PS postop^a^	1.11	1.07–1.14	1.11	1.07–1.14
Work tasks				
Physical (ref = mental)	0.15	0.04–0.60	0.20	0.06–0.69
Both (ref = mental)	0.14	0.04–0.46	0.64	0.20–2.30

Adjusted for preoperative expectation

^a^Scales ranged from 0 to 100; higher scores indicated better outcomes

*HOOS-PS* Hip injury and Osteoarthritis Outcome Score – Physical function Short form, *KOOS-PS* Knee injury and Osteoarthritis Outcome Score—Physical function Short form, *Ref* reference category, *Postop* postoperatively

### Expectation fulfilment of TKA patients

Six months postoperatively, 72% (n = 211) of patients had fulfilled expectations, with 8% (n = 17) exceeding their expectations; this percentage increased to 79% (n = 204) and 9% (n = 18), respectively, at 12 months postoperatively. Six months postoperatively, 81% of patients with unfulfilled and 95% of patients with fulfilled expectations actually returned to work, increasing 12 months postoperatively to 85% and 98%, respectively. Preoperatively, mean expected time to RTW was 11 weeks for patients with unfulfilled and 9 weeks for patients with fulfilled expectations. Actual mean time to RTW was 13 weeks for patients with unfulfilled and 11 weeks for patients with fulfilled expectations.

Potential risk factors stratified for fulfilment are shown in Tables S1 and S2. Six months postoperatively, seven factors were below the cut-off value in the univariate analyses and therefore used in the multivariate analyses (age, musculoskeletal comorbidity, preoperative KOOS-PS, postoperative KOOS-PS, work tasks, preoperative sick leave, postoperative difficulties at work due to knee complaints; Table S3). Higher age (OR 1.08, 95% CI 1.01–1.15) and better postoperative physical functioning (KOOS-PS) scores (OR 1.03, 95% CI 1.01–1.06) increased the likelihood of fulfilment. Preoperative sick leave (reported in 31%; OR 0.33, 95% CI 0.17–0.65) and postoperative difficulties at work due to knee complaints (reported in 56%; OR 0.41, 95% CI 0.17–0.98) decreased the likelihood of fulfilment (Table 2). Sensitivity analyses showed that a lower proportion of patients aged ≤ 55 had fulfilled expectations compared to those aged ≥ 56.

Twelve months postoperatively, six factors were below the cut-off value in the univariate analyses (musculoskeletal comorbidity, preoperative KOOS-PS, postoperative KOOS-PS, work tasks, preoperative sick leave, postoperative difficulties at work due to knee complaints; Table S4). Better postoperative physical functioning (OR 1.11. 95% CI 1.07–1.14) increased the likelihood of fulfilment. Physical work tasks (OR 0.20, 95% CI 0.06–0.69) decreased the likelihood of fulfilment (Table 3).

## Discussion

This study investigated factors associated with fulfilment of patient expectations towards paid employment after THA or TKA at 6 and 12 months postoperatively. Six months after THA, 75% of patients had fulfilled expectations, increasing to 83% at 12 months postoperatively. Six months after TKA, 72% of patients had fulfilled expectations, increasing to 79% at 12 months postoperatively. Preoperative factors associated with fulfilment were older age, mental work tasks (compared to physical work tasks), and no sick leave due to knee complaints (only at 6 months postoperatively). Postoperative factors associated with fulfilment were better physical functioning, and no difficulties at work due to hip or knee complaints (only at 6 months postoperatively).

In our study, the majority of patients had high preoperative expectations towards paid employment, which is in line with the results of previous studies among THA and TKA patients [5, 9, 27]. The proportion of fulfilled expectations towards paid employment we found is also in line with previous literature among THA and TKA patients [10, 11]. Patient-specific education might create more realistic expectations on outcome, resulting in satisfaction and better postoperative outcomes of THA/TKA patients, and thus fulfilment of preoperative expectations [9, 28, 29].

We found that older age was associated with fulfilment 6 months after TKA and 12 months after THA. In-depth analyses showed that a lower proportion of patients aged ≤ 55 had fulfilled expectations compared to those aged ≥ 56. Results on the association between age and fulfilment among arthroplasty patients are conflicting [11, 30]. One study among TKA patients did not find an association [11], another study suggested that younger age of only THA patients, but not TKA patients, was associated with general fulfilment [30]. The contradictory results could be attributed to inclusion of all age groups, resulting in a different age distribution and also in a higher average age. Differences in methods (measurement of fulfilment and investigating overall or general fulfilment) and measurements at only 12 months postoperatively could also account for the contradiction.

Our results showing that better postoperative physical functioning after both THA and TKA was associated with fulfilled expectations, were in accordance with studies focusing on overall or general fulfilment after THA or TKA [10, 30].

Our study showed that preoperative sick leave decreased the likelihood of fulfilment after TKA at 6 months postoperatively. Patients who performed mainly physical tasks were less likely to have fulfilled expectations (THA at 6 and 12 months postoperatively, TKA at 12 months postoperatively). Also, both THA and TKA patients with postoperative difficulties at work due to hip or knee complaints were less likely to have fulfilled expectations 6 months postoperatively. These results could not be compared to previous studies about expectation fulfilment, therefore further research is needed to confirm our results. Still, it is known that these factors also influence RTW outcomes after THA and TKA [31–35].

In a previous study on sex differences in expectations after THA and THA we found that both preoperative expectations and their fulfilment was higher in men then in women [36]. In our current study sex was not identified as a risk factor for fulfilment for paid employment. The conflicting results are probably due to the difference in study population, our study only included working-age patients with preoperative paid employment whereas the previous study also included older patients (age > 64) and without preoperative paid employment.

### Implications

Patients beliefs and expectations have been linked to RTW among THA and TKA patients [37–39]. However, being returned to work does not inevitably mean that patients experience good work functioning. Hence, it is important to also look beyond RTW and focus on fulfilment of expectations towards paid employment and associated factors. Orthopaedic surgeons could use the results in the shared-decision making process for THA/TKA, to preoperatively manage patient expectations, and to assess whether additional RTW guidance is necessary. Further research is needed to unravel fulfilment of expectations towards paid employment. Furthermore, the results show that some factors influencing fulfilment may be modifiable (i.e. work tasks, difficulties at work due to hip or knee complaints) and could be targeted by the occupational physician or the employer. Our results thus suggest that orthopaedic surgeons, occupational physicians and employers may contribute to address those patients prone to unfulfillment.

### Strengths and limitations

A strength of this study is its design, with outcome measures at both 6 and 12 months postoperatively and a relatively large sample size. Also, our study population resembles the population in the Dutch Arthroplasty Register (LROI) based on ASA-classification and BMI [6]. The proportion of females in our study was lower compared to the Dutch Arthroplasty Register, which might be the result of fewer females having paid work in general. However, in the LOAS the proportion females is the same as in the Dutch Arthroplasty Register, therefore, generalizability of the results to the Dutch arthroplasty population is another strength.

Study limitations included the self-reported data used in the study. BMI was derived from self-reported body height and weight which may have introduced extra measurement error. The assessment of patient expectations and their fulfilment, which was measured with only one item of the validated HSS questionnaire. However, there is no questionnaire specifically focussing on expectations towards paid employment. The HSS questionnaire was originally developed to assess preoperative expectations [25], yet other studies have used the same approach to determine postoperative fulfilment [10, 12, 30]. The majority of patients who were lost to follow-up were male and performed mainly mental work tasks. Male sex has previously been linked to loss to follow-up [40]. The loss to follow-up of patients with mainly mental work tasks might have diluted our results to some extent. Last, it remains unknown why patients answered “not applicable”, since they all were of working-age and had a paid job preoperatively. The majority of these patients performed mainly mental work tasks (THA 56%; TKA 43%) and a lower proportion was preoperatively on sick leave (THA 9%; TKA 9%). It could be that these patients had no expectations or expected deterioration, or that their osteoarthritis complaints did not affect their work and therefore had no expectations.

## Conclusions

This study illustrates that only three out of four THA or TKA patients have fulfilled expectations 6 months after surgery. Older age, mental work tasks, no preoperative sick leave, better postoperative physical functioning, and no postoperative difficulties at work were identified as factors that increased the likelihood of fulfilment of patient expectations towards paid employment after THA or TKA. Further quantitative and qualitative research is necessary to explore which factors influence patient expectation fulfilment towards paid employment, to eventually design and implement effective targeting strategies to appropriately address and support those prone to unfulfillment, and to better match preoperative expectations and postoperative fulfilment after THA or TKA.

### Supplementary Information

Below is the link to the electronic supplementary material.

Supplementary file1 (DOCX 57 KB)

## Data Availability

The data underlying this article were provided by the LOAS study group with permission. The data will be shared upon reasonable request to the corresponding author, with permission of the LOAS study group.
