# Supplementary material for: Three out of four working-age patients have fulfilled expectations towards paid employment six months after total hip or knee arthroplasty: a multicentre cohort study
Source: Rheumatol Int. 2023 Aug 29;44(2):339–47. doi: 10.1007/s00296-023-05437-9 (PMC10796735; doi:10.1007/s00296-023-05437-9)
Supplement: Supplementary file 1 — Supplementary file1 (DOCX 57 KB) [file 296_2023_5437_MOESM1_ESM.docx]

**Fulfilment of expectations towards paid employment after total hip or knee arthroplasty**

Journal: Rheumatology International

Authors: Tamara Kamp^a,b,c^, Martin Stevens^a^, Thea P.M. Vliet Vlieland^c^, Rob G.H.H. Nelissen^c^, Sandra Brouwer^b^, Maaike G.J. Gademan^c,d^ on behalf of the Longitudinal Leiden Orthopaedics Outcomes of Osteoarthritis Study (LOAS) Group

^a^ Department of Orthopaedics, University of Groningen, University Medical Center Groningen, Groningen, The Netherlands

^b^ Department of Health Sciences, Community and Occupational Medicine, University of Groningen, University Medical Center Groningen, Groningen, The Netherlands

^c^ Department of Orthopaedics, Leiden University Medical Center, Leiden, The Netherlands

^d^ Department of Clinical Epidemiology, Leiden University Medical Center, Leiden, The Netherlands

*Address for correspondence and preprint requests:*

Tamara Kamp
Department of Orthopaedics

University Medical Center Groningen, University of Groningen
P.O. Box 30.001
9700 RB Groningen, The Netherlands
E-mail: t.kamp@umcg.nl

**Table S1:** Analyses for the outcome postoperative fulfilment of expectations towards paid employment 6 months after total hip arthroplasty (THA) or total knee arthroplasty (TKA)

|  | **THA (N=316)** | | | **TKA (N=294)** | | |
| --- | --- | --- | --- | --- | --- | --- |
| **Expectations towards paid employment** | **Unfulfilled**  **(n=79, 25%)** | **Fulfilled/exceeded**  **(n=237, 75%)** | **P-value** | **Unfulfilled**  **(n= 83, 28%)** | **Fulfilled/exceeded**  **(n=211, 72%)** | **P-value** |
| *Sociodemographic factors* |  |  |  |  |  |  |
| Age (years), mean (SD) | 56 (6) | 57 (4) | 0.22 | 57 (5) | 58 (4) | 0.00 |
| Sex (female) | 56% | 47% | 0.19 | 63% | 55% | 0.26 |
| *Health-related factors* |  |  |  |  |  |  |
| Comorbidity   - Musculoskeletal (yes) - Non-musculoskeletal (yes) | 56%  56% | 47%  51% | 0.19  0.47 | 51%  66% | 39%  61% | 0.07  0.35 |
| HOOS-PS/KOOS-PS preop, mean (SD) | 42 (16) | 45 (18) | 0.14 | 44 (8) | 42 (16) | 0.05 |
| HOOS-PS/KOOS-PS 6 months postop**^a^**, mean (SD) | 68 (15) | 86 (13) | 0.00 | 57 (18) | 68 (13) | 0.00 |
| *Work-related factors* |  |  |  |  |  |  |
| Work tasks   - Physical - Mental - Both | 25%  19%  56% | 17%  43%  39% | 0.00 | 29%  13%  58% | 19%  31%  51% | 0.00 |
| Sick leave 1month preop (yes) | 42% | 22% | 0.00 | 45% | 24% | 0.00 |
| Difficulties at work due to hip/knee 6 months postop (yes) | 79% | 35% | 0.00 | 71% | 55% | 0.00 |
| RTW (yes) | 82% | 96% | 0.00 | 81% | 95% | 0.00 |
| Expected time to RTW (weeks), mean (SD) | 11 (7) | 7 (5) | 0.00 | 11 (5) | 9 (6) | 0.01 |
| Actual time to RTW (weeks), mean (SD) | 12 (5) | 9 (5) | 0.00 | 13 (8) | 11 (5) | 0.06 |

*Data are presented as mean with standard deviation (SD), or as percentages.*

*^a^Scales ranged from 0 to 100; higher scores indicated better outcomes.*

*HOOS-PS = Hip injury and Osteoarthritis Outcome Score – Physical function Short form; KOOS-PS = Knee injury and Osteoarthritis Outcome Score – Physical function Short form; preop = preoperatively; postop = postoperatively; RTW = return to work.*

**Table S2:** Analyses for the outcome postoperative fulfilment of expectations towards paid employment 12 months after total hip arthroplasty (THA) or total knee arthroplasty (TKA)

|  | **THA (N=287)** | | | **TKA (N=258)** | | |
| --- | --- | --- | --- | --- | --- | --- |
| **Expectations towards of paid employment** | **Unfulfilled**  **(n=49, 17%)** | **Fulfilled/exceeded**  **(n=238, 83%)** | **P-value** | **Unfulfilled**  **(n=54, 21%)** | **Fulfilled/exceeded**  **(n=204, 79%)** | **P-value** |
| *Sociodemographic factors* |  |  |  |  |  |  |
| Age (years), mean (SD) | 54 (7) | 57 (6) | 0.01 | 58 (4) | 58 (5) | 0.93 |
| Sex (female) | 53% | 48% | 0.46 | 51% | 56% | 0.60 |
| *H*ealth-related *factors* |  |  |  |  |  |  |
| Comorbidity   - Musculoskeletal (yes) - Non-musculoskeletal (yes) | 61%  59% | 45%  51% | 0.04  0.28 | 52%  59% | 40%  60% | 0.12  0.94 |
| HOOS-PS/KOOS-PS preop**^a^**, mean (SD) | 39 (19) | 46 (17) | 0.03 | 34 (18) | 43 (15) | 0.00 |
| HOOS-PS/KOOS-PS 12 months postop**^a^**, mean (SD) | 70 (15) | 89 (11) | 0.00 | 56 (14) | 73 (13) | 0.00 |
| *Work-related factors* |  |  |  |  |  |  |
| Work tasks   - Physical - Mental - both | 25%  12%  63% | 14%  43%  42% | 0.00 | 37%  13%  48% | 18%  24%  58% | 0.01 |
| Sick leave 1month preop (yes) | 37% | 21% | 0.02 | 52% | 25% | 0.00 |
| Difficulties at work due to hip/knee 12 months postop (yes) | 67% | 18% | 0.00 | 44% | 22% | 0.00 |
| RTW (yes) | 88% | 98% | 0.00 | 85% | 98% | 0.00 |
| Expected time to RTW (weeks), mean (SD) | 11 (7) | 8 (4) | 0.00 | 13 (10) | 10 (6) | 0.03 |
| Actual time to RTW (weeks), mean (SD) | 15 (13) | 11 (12) | 0.17 | 13 (5) | 12 (6) | 0.44 |

*Data are presented as mean with standard deviation (SD) or as percentages.*

*^a^Scales ranged from 0 to 100; higher scores indicated better outcomes.*

*HOOS-PS = Hip injury and Osteoarthritis Outcome Score – Physical function Short form; KOOS-PS = Knee injury and Osteoarthritis Outcome Score – Physical function Short form; preop = preoperatively; postop = postoperatively; RTW= return to work.*

**Table S3.** Univariate regression analyses for the outcome postoperative fulfilment of expectations towards paid employment 6 months after total hip arthroplasty (THA) and total knee arthroplasty (TKA)

|  | **THA** | | | **TKA** | | |
| --- | --- | --- | --- | --- | --- | --- |
| **Variables** | **OR** | **95% CI** | **P-value** | **OR** | **95% CI** | **P-value** |
| Age (years) | 1.03 | 0.99–1.07 | 0.21 | 1.09 | 1.03–1.15 | 0.00 |
| Sex (ref = male) | 0.71 | 0.43–1.19 | 0.20 | 0.74 | 0.44–1.26 | 0.27 |
| Musculoskeletal comorbidity (ref = no) | 0.72 | 0.43–1.21 | 0.22 | 0.62 | 0.37–1.03 | 0.06 |
| Non-musculoskeletal comorbidity (ref = no) | 0.85 | 0.51–1.42 | 0.52 | 0.77 | 0.45–1.32 | 0.35 |
| HOOS-PS / KOOS-PS preop^a^ | 1.01 | 1.00–1.03 | 0.14 | 1.02 | 1.00–1.03 | 0.05 |
| HOOS-PS / KOOS-PS postop^a^ | 1.10 | 1.07–1.13 | 0.00 | 1.05 | 1.03–1.07 | 0.00 |
| Work tasks |  |  |  |  |  |  |
| - physical (ref = mental) | 0.31 | 0.14–0.66 | 0.00 | 0.27 | 0.12–0.62 | 0.00 |
| - Both (ref = mental) | 0.32 | 0.17–0.61 | 0.00 | 0.38 | 0.19–0.79 | 0.01 |
| Sick leave preop (ref=no) | 0.39 | 0.22–0.67 | 0.00 | 0.37 | 0.21–0.63 | 0.02 |
| Difficulties at work due to hip/knee postop (ref=no) | 0.06 | 0.02–0.14 | 0.00 | 0.22 | 0.10–0.47 | 0.00 |

*Adjusted for preoperative expectation.*

*^a^All scales ranged from 0 to 100; higher scores indicated better outcomes*

*HOOS-PS = Hip injury and Osteoarthritis Outcome score – Physical function Short form; KOOS-PS = Knee injury and Osteoarthritis Outcome Score - Physical function Short form; Ref= reference category; preop = preoperatively; postop = postoperatively.*

**Table S4.** Univariate regression analyses for the outcome postoperative fulfilment of expectations towards paid employment 12 months after total hip arthroplasty (THA) and total knee arthroplasty (TKA)

|  | **THA** | | | **TKA** | | |
| --- | --- | --- | --- | --- | --- | --- |
| **Variables** | **OR** | **95% CI** | **P-value** | **OR** | **95% CI** | **P-value** |
| Age (years) | 1.06 | 1.02–1.11 | 0.01 | 1.00 | 0.93–1.07 | 0.97 |
| Sex (ref = male) | 0.81 | 0.44–1.51 | 0.51 | 1.23 | 0.67–2.25 | 0.51 |
| Musculoskeletal comorbidity (ref = no) | 0.51 | 0.27–0.97 | 0.04 | 0.63 | 0.34–1.16 | 0.14 |
| Non-musculoskeletal comorbidity (ref = no) | 0.71 | 0.38–1.32 | 0.27 | 1.03 | 0.56–1.91 | 0.92 |
| HOOS-PS / KOOS-PS preop^a^ | 1.02 | 1.00–1.04 | 0.02 | 1.04 | 1.02–1.06 | 0.00 |
| HOOS-PS / KOOS-PS postop^a^ | 1.10 | 1.07–1.13 | 0.00 | 1.10 | 1.07–1.14 | 0.00 |
| Work tasks |  |  |  |  |  |  |
| - Physical (ref = mental) | 0.17 | 0.06–0.48 | 0.00 | 0.27 | 0.10–0.70 | 0.01 |
| - Both (ref = mental) | 0.18 | 0.07–0.46 | 0.00 | 0.65 | 0.26–1.61 | 0.35 |
| Sick leave preop (ref=no) | 0.45 | 0.23–0.88 | 0.02 | 0.29 | 0.16–0.55 | 0.00 |
| Difficulties at work due to hip/knee postop (ref=no) | 0.06 | 0.03–0.16 | 0.00 | 0.17 | 0.06–0.48 | 0.00 |

*Adjusted for preoperative expectation.*

*^a^All scales ranged from 0 to 100; higher scores indicated better outcomes*

*HOOS-PS = Hip injury and Osteoarthritis Outcome Score – Physical function Short form; KOOS-PS = Knee injury and Osteoarthritis Outcome Score - Physical function Short form; Ref = reference category; preop = preoperatively; postop = postoperatively.*
